# Environmental context effects on affective and cognitive empathy in virtual reality

**DOI:** 10.1038/s41598-026-66551-9

**Published:** 2026-08-14

**Authors:** Till Kastendieck, Daniel Huppertz, Heidi Mauersberger, Ursula Hess

**Affiliations:** 1 Department of Psychology, Humboldt University of Berlin, Berlin, Germany; 2 Berlin School of Mind and Brain, Humboldt University of Berlin, Berlin, Germany

**Keywords:** Emotion communication, Affective empathy, Cognitive empathy, Emotional mimicry, Embeddedness, Situatedness, Environmental context, Virtual reality, Ecological validity, Neuroscience, Psychology, Psychology

## Abstract

Human interaction invariably occurs within a specific context. Although recent research highlights the role of context for emotion communication, the specific influence of the environmental *ambience* (the setting’s atmosphere) is less understood. This study investigated the influence of positive versus negative ambience on both cognitive (emotion recognition) and affective empathy (emotional mimicry). In a laboratory experiment, 75 participants, wearing head-mounted displays with facial EMG sensors, encountered avatars expressing joy or distress (sadness, anger, and disgust) within either pleasant or unpleasant immersive ambiences. As predicted, mimicry was stronger when the expressed emotion matched the ambience. Furthermore, positive ambience enhanced cognitive empathy for sad and happy expressions. These findings show that the ambience of an encounter significantly shapes our empathetic responses to others within that environment.

**Supplementary Information:**

The online version contains supplementary material available at 10.1038/s41598-026-66551-9.

## Introduction

Douglas Adams famously noted, “It can hardly be a coincidence that no language on Earth has ever produced the expression ‘as pretty as an airport’.” He then elaborates on the unsettling emotional impact of airports. This example illustrates the notion that humans have affective responses to their surroundings that may impact on how we react to others.

When we encounter others we evaluate our own position relative to the other person (e.g.,^1^) as well as their potential intentions^2,3^, but also the nature of the situation^4,5^. These factors all impinge on how we react during the encounter. However, while there is a burgeoning literature on the impact of social context factors such as group membership^6,7^ or perceived closeness^8,9^, but also scene-face matches^10^ or between-channel congruence^11^ on emotion communication, the environmental setting in which an encounter takes place has received little attention. This lack may in part be due to the difficulty of placing participants into a given environmental setting while obtaining fine-grained measures of their emotional behavior. VR technology makes this possible but can interfere with the measurement of facial reactions. In this study, we used a unique combination of high-resolution VR technology and facial electromyography (EMG) to address the question of how people perceive and react to others in pleasant versus unpleasant environmental settings. Specifically, we focused on the effect of environmental settings on empathic reactions to others.

### Empathy

Empathy can be defined as the ability and tendency to share and understand others’ internal states^12^. The former is often referred to as affective empathy and the latter as cognitive empathy (see^13,14^). Thus, empathy comprises the bottom-up process of emotional resonance^15^ and the top-down processes of understanding. In the present study, we assessed cognitive empathy through emotion ratings and affective empathy through emotional mimicry^16–20^.

### Emotional mimicry

Emotional mimicry – the matching of an interaction partner’s emotional display -- is an ubiquitous process that can be readily observed in everyday life^16^. Emotional mimicry is not restricted to humans and may even be a common response in species that display emotions. It has been observed, amongst others, in non-human primates (e.g.,^21,22^), but also dogs^23^ and dolphins^24^, most commonly in situations involving play. The *mimicry as social regulator model*^17,18^ considers human mimicry a social act and as such influenced by the social context of the interaction and the social goals of the mimicker. It posits that mimicry is automatic but goal dependent. The goals that are served by mimicry are to communicate with others in order to foster affiliation and to regulate interpersonal closeness. Since emotional mimicry depends crucially on affiliation, it can be used as an index for the affiliative intent of the mimicker and thereby serve as a proxy for affective empathy^20,25^.

### The effect of positive versus negative ambience on emotion communication

To allow for a clearer distinction between context as emotion elicitor and context as a “setting” the term *ambience* is useful. Merriam-Webster defines *ambience* as a feeling or mood associated with a particular place, person, or thing^26^. That is, the setting or ambience of a place in which the observer is immersed elicits a mood state in the observer. This mood is then expected to impact on the empathic reactions towards others. As such, the environmental setting or ambience in which an interaction takes place is different from the specific emotion elicitors that elicit an emotion in the expresser. When for example, two people are meeting for a first date in a park it is most likely not the park that makes them smile. Nonetheless, the positive mood induced by the park may have an impact on their smiles.

Specifically, the broaden-and-build theory^27,28^ posits that positive affect expands our interest, awareness, and options for action (i.e., “momentary thought-action repertoires”,^27^, * p. 218*), thereby supporting the development of social and interpersonal resources. In contrast, negative states tend to narrow focus and constrain behavioral repertoires^28,29^.

This leads to the prediction that a pleasant ambience facilitates social interaction and fosters empathy in terms of emotion recognition and emotional mimicry, as well as perceived interpersonal closeness. By contrast, an unpleasant ambience should thwart these processes. As such, positive ambiences can be said to provide the resources for more empathy with others (henceforth, empathy resources hypothesis).

The contexts studied to date with regard to emotion communication are quite diverse and range from situational context, which describes the circumstances surrounding an expressive event^30^, or the concurrent presentation of a facial expression and the purported elicitor of the expression^31^ to disembodied faces superimposed on scenes^10^, and include even other nonverbal channels such as postures^11^. Notably, in much of this line of research, context as emotion elicitor and context as “setting” tends to be confounded as participants typically see scene-face compounds where the scenes implicitly or explicitly serve as emotion elicitor for the expressions shown.

That said, the impact of contexts in this wider sense on emotion perception^32,33^, person perception^34^ and emotional mimicry^18^ can be considered as well-established. Research that focused on scenic contexts highlights that contexts incongruent with the emotion shown can interfere with both emotion perception (e.g.,^10^) and mimicry^8^. Moreover, face-scene compounds are processed faster and more accurately when they are valence-congruent rather than incongruent^35^. Based on these findings, we would predict more mimicry and better emotion recognition for congruent compared to incongruent settings (henceforth, congruency hypothesis).

### Virtual reality

One important aspect for the study of ambience is that for a context to be ambient, the perceiver has to be immersed in it and feel present. Still photographs and even videos are less likely to provide this sense of being *in* a situation rather than just perceiving it from afar. Virtual reality (VR) offers a promising solution to this problem. VR can enhance realism, immersion, and the associated feeling of presence while allowing control over experimental conditions. As such, it can be used to create immersive environments that affect mood states^36^ as required for the study of ambience. Yet one challenge in the context of mimicry research is that the emotional mimicry of facial expressions involves the face, which is usually obscured by VR headsets. Also, mimicry is often quite subtle, hence, facial electromyography (EMG) is the measure of choice^37^. But affixing electrodes under a high-resolution VR headset can be challenging and may cause issues with headset fit and signal quality^38^. In the present study, this problem was solved by the use of a VR headset with built-in electrodes (emteqPRO^39^).

### Overview of the study

**Fig. 1:**
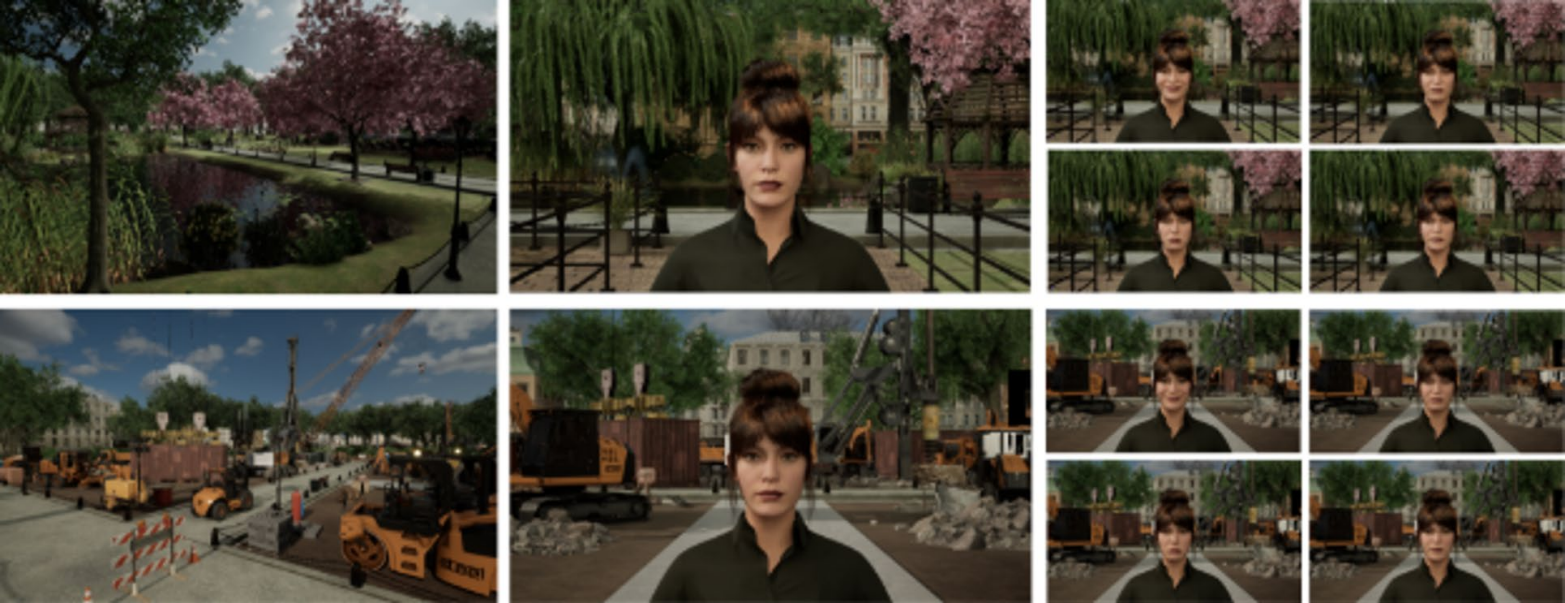
Still frames of VR environments and avatar expressions. Far left: pleasant (top) and unpleasant (bottom) place; center: neutral expression in pleasant (top) and unpleasant (bottom) place; right: joy, sadness, anger, disgust (from top left to right bottom). Avatar expressions on the right recorded at apex.

The present research investigated the effect of ambience on emotion communication. For this, a high-definition VR setup was used that allowed participants to be immersed in an audio-visual scene depicting either a pleasant park area with greenery or a construction site within the park (see Fig. 1). Participants virtually “walked” around the park and experienced its ambience. At different locations in the virtual park, they encountered avatars, who showed different facial expressions. Given that in typical everyday settings, we encounter more people who smile than frown^40^ half the expressions were smiles whereas the remainder were an even mix of sad, disgusted and angry expressions, that we collectively refer to as distress.

We predicted that participants’ mimicry of the avatar’s expression would be impacted by the ambience of the environment. For happy expressions, the congruency and empathy resources hypotheses converge in predicting better recognition and stronger emotional mimicry in a pleasant ambience. For distressed expressions, however, the two accounts diverge. The congruency hypothesis predicts better recognition and stronger mimicry in the matching unpleasant ambience, whereas the empathy resources hypothesis^28^ predicts that the pleasant ambience may facilitate empathic responding even to negative expressions. This would also be in line with the general stance of Hess and Fischer^16^, that perceivers are more likely to mimic if they are motivated to affiliate with (and feel psychologically close to) the mimickee, which seems more likely in a positive environment. The study design and hypotheses were pre-registered via the *Open Science Framework*.

## Results

### Data analysis considerations

We analyzed the data using ANOVA style linear mixed modeling (LMM with lmerTest^41^), with the fixed factors facial expression and ambience and the random factor participant id. Post-hoc tests were conducted with *emmeans*^42^ in R^43^ version 4.2.1.

To assess mimicry, mean EMG was analyzed across four time points. Neutral: when the avatar is first encountered and shows a neutral expression (1.5 s); onset: the dynamic onset period of the expression (2 s), apex: the maximum intensity of the expression (1.5 s) and poststim: the time period of 1.5 s following the disappearance of the face.

Facial expressions are patterns. Hence it is important to not only focus on the activity of single muscles but rather consider the pattern of muscle activity^37^. Hence, mimicry was considered present when a contrast comparing activity of Corrugator S. with the mean of O. Oculi and Zygomaticus M. (Helmert contrast) was significant. Differences in mimicry as a function of ambience were evaluated by the a-priori comparison of the contrast means across the two ambiences. Due to the complexity of the full model, which caused convergence issues, mimicry was evaluated separately for happy and distress expressions.

Emotion recognition was assessed by comparing the rating on the target scale (i.e., happy for happy expressions) with all other ratings. This was done separately for each specific expression (i.e., happy, sad, angry and disgusted). Differences in emotion recognition as a function of ambience were evaluated by the a-priori comparisons of scale ratings across the two ambiences.

### Manipulation check

Following the experimental tasks, participants were asked to report on their emotions with regard to the visual and auditory elements of the pleasant and unpleasant ambiences on positive and negative emotion scales ranging from 1- not at all to 101- very intensely. We combined ratings of happiness and surprise into a positive emotion scale and ratings of anger, sadness, fear, and disgust into a negative emotion scale (see Table 1 for means, SDs and 95% confidence intervals).

**Table 1:** Means, SDs and 95% confidence intervalls for manipulation check ratings

Ambience	Positive emotion			Negative emotion		
	Mean	SD	95% CI	Mean	SD	95% CI
Visual						
Pleasant	45.20	19.47	40.81,49.59	2.96	4.76	1.88, 4.03
Unpleasant	9.38	12.81	6.49,12.26	19.45	14.41	16.20, 22.70
Auditory						
Pleasant	38.60	20.90	33.89,43.31	5.20	12.55	2.37, 8.02
Unpleasant	7.46	9.92	5.22,9.69	21.70	15.40	18.23, 25.18

An LMM with the factors Ambience (pleasant, unpleasant), Emotional valence (positive, negative) and Context (visual, auditory) confirmed that participants felt that both the visual and the auditory context elicited more positive emotion (t_visual_ = 14.49, *p* < .001, d = 0.34; t_auditory_ = 12.61, *p* < .001, d = 0.30) and less negative emotion (t_visual_ = 9.45, *p* < .001, d = 0.22; t_auditory_ = 9.44, *p* < .001, d = 0.22) in the pleasant ambience context compared to the unpleasant context (for the full analyses see supplementary materials).

### Emotional mimicry

#### Happy expressions

**Fig. 2:**
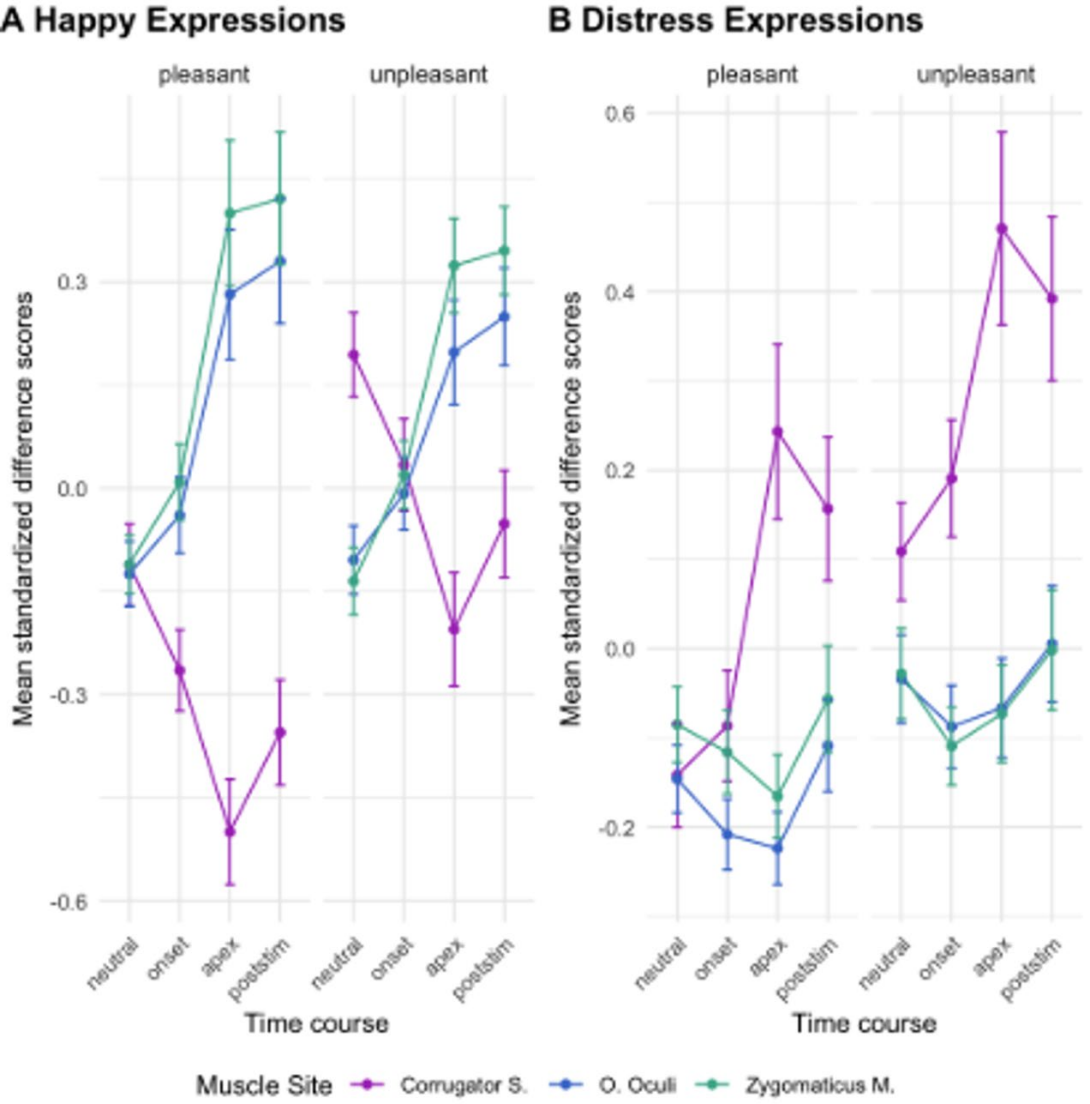
Facial EMG means and standard errors as a function of time, muscle site and ambience for happy (**A**) and distress (**B**) expressions.

Significant main effects of time, F(3, 9846) = 58.25, *p* < .001, h_p_^2^ = 0.017, muscle site, F(2, 9870) = 232.00, *p* < .001, eta_p_^2^ = 0.045, ambience, F(1, 9916) = 22.27, *p* < .001, eta_p_^2^ = 0.002, were qualified by time x muscle site, F(6, 9845) = 79.72, *p* < .001, h_p_^2^ = 0.046, and muscle site x ambience interactions, F(2, 9850) = 59.41, *p* < .001, eta_p_^2^ = 0.012. Means and standard errors are shown in Fig. 2.

We assessed whether expressions were mimicked by evaluating a Helmert contrast comparing activity of the Corrugator S. with the mean activity of O. Oculi and Zygomaticus M. at each of the four time periods for pleasant and unpleasant ambience.

For *pleasant* ambience, the contrast was significant for onset, apex, and the 1.5s-period after stimulus offset (poststim). For *unpleasant* ambience the contrast was also significant at neutral when no expression was shown. The direction of the contrast indicates that Corrugator S. was more active than O. Oculi and Zygomaticus M. indexing a negative expression indicative of mood induction by the ambient context. A significant contrast indicative of a positive expression was present for apex and poststim.

A-priori comparisons of the mean contrast score across ambience conditions were significant for all four time points. Of relevance here are the two times points where we found mimicry in both conditions. Overall, as predicted, a more intense mimicry reaction was shown in the pleasant ambience (apex t = 5.49, *p* < .001; poststim t = 6.04, *p* < .001). In the unpleasant ambience, emotion induction through the ambience conflicted with mimicry in the early stages. For more detail see supplementary materials.

#### Distress expressions

Significant main effects of time, F(3, 10362) = 19.77, *p* < .001, eta_p_^2^ = 0.005, muscle site, F(2, 10383) = 214.94, *p* < .001, eta_p_^2^ = 0.040, ambience, F(1, 10434) = 139.24, *p* < .001, eta_p_^2^ = 0.013, were qualified by time x muscle site, F(6, 10362) = 21.95, *p* < .001, eta_p_^2^ = 0.013, and muscle site x ambience interactions, F(2, 10367) = 27.47, *p* < .001, eta_p_^2^ = 0.005. Means and standard errors are shown in Fig. 2.

Helmert contrasts across the four time periods showed that in the *pleasant* ambience participants showed a significantly negative facial expressive pattern (congruent with mimicry of negative emotions) during apex and poststim. The contrast just failed to be significant for onset (*p* = .051). In the *unpleasant* ambience condition, the contrast was significant at all four time periods, indicating that participants already showed a negative expression while watching the avatar with a neutral expression. This again suggests the persistent presence of negative affect induction. Notably however, the negative facial expression then intensified in reaction to the avatar’s expression. By contrast, for *pleasant* ambience affect induction did not seem to persist as strongly but the reaction was slowed down as mimicry was not significantly present at onset. The mean contrast was significantly different across the two ambience conditions for all four time points with stronger mimicry in the congruent condition (apex t = 3.12, *p* = .002; poststim t = 3.33, *p* = .001). For more detail on the analysis and post-hoc tests see supplementary materials.

In sum, both happy and distress faces were mimicked, and ambience had a strong effect on both expressions. In line with the *congruency hypothesis*, mimicry was dampened in the incongruent conditions. Notably, in a negative ambience environment, mimicry seems to conflict with emotion expressions induced by ambience as these expressions persisted into the initial contact with the avatar as shown by participants’ negative facial expressions during the neutral face presentation. Positive ambience did not lead to the same persistence in facial affect, even though the manipulation-check suggested that positive affect induction was somewhat stronger than negative affect induction.

### Emotion recognition

**Fig. 3:**
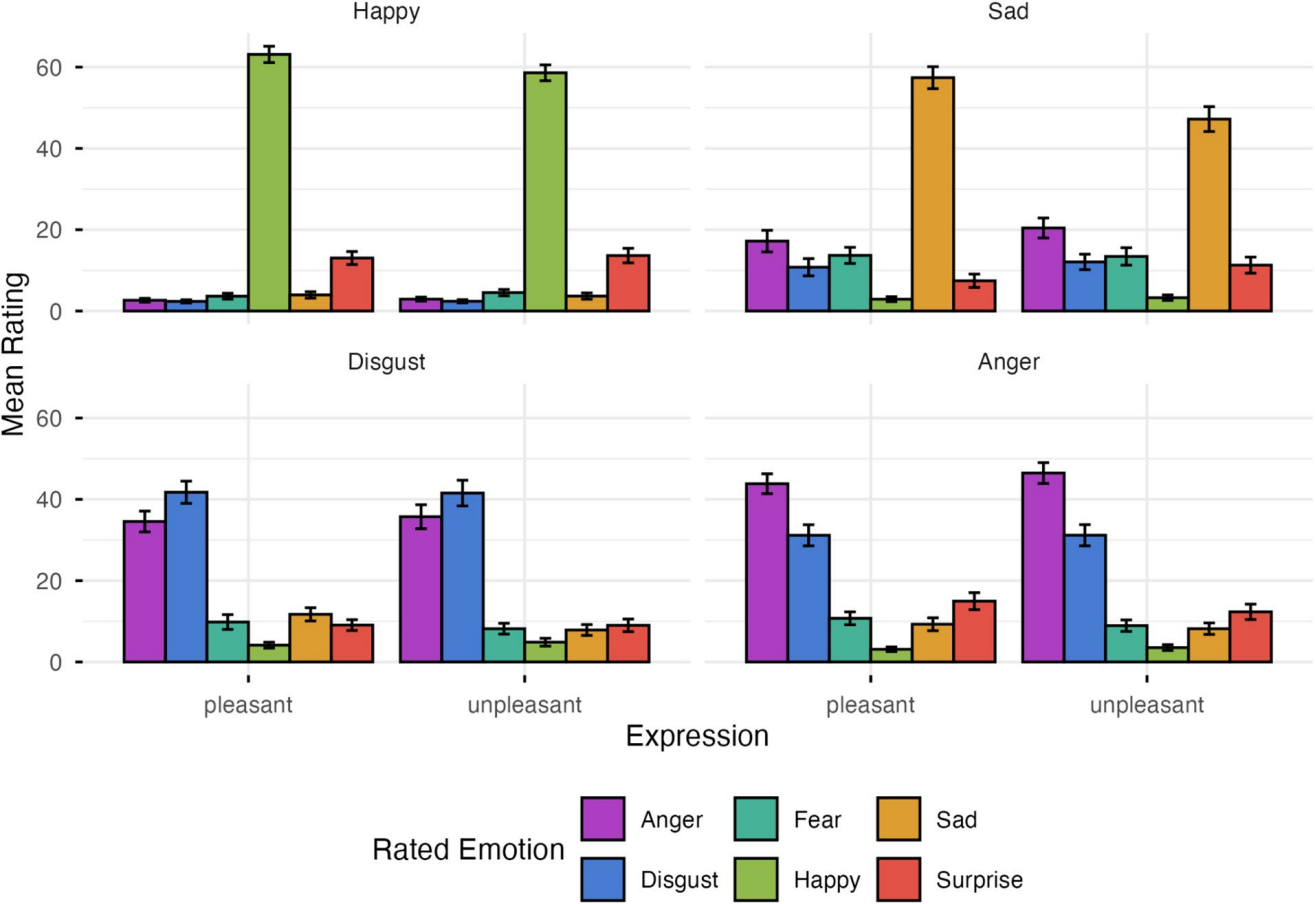
Means and standard errors for emotion ratings as a function of expression and ambience.

While we expected mimicry reactions to be restricted to positive vs. negative expressions^16^, emotion recognition needs to be assessed as a function of the discrete emotion shown (e.g., happy, sad, disgusted, angry). An LMM with the factors Emotion scale (happiness, sadness, anger, fear, disgust, surprise), Expression type (happy, sad, disgusted, angry), and Ambience, revealed significant main effects of Scale, F(5, 9796) = 228.18, *p* < .001, eta_p_^2^ = 0.104 and Expression, F(3, 9830) = 51.04, *p* < .001, eta_p_^2^ = 0.015, which were qualified by a Scale by Ambience, F(5, 9796) = 5.18, *p* < .001, eta_p_^2^ = 0.003, and a Scale by Expression interaction, F(15, 9796) = 859.83, *p* < .001, eta_p_^2^ = 0.568, as well as the three-way interaction, F(15, 9796) = 3.14, *p* < .001, eta_p_^2^ = 0.005. For means and standard errors see Fig. 3. To follow up on the three-way interaction, we assessed for each expression (a) whether the target emotion (e.g., happiness for happy expressions) was rated most intensely on the emotion profile; and (b) whether ambience affected any of the emotion ratings in the profile.

For all expressions, pairwise contrasts confirmed that the target emotion rating was highest across all emotions. These findings also serve as manipulation check for the expressions shown by the avatars and confirm that the expressions were perceived as intended. For anger and disgust expressions both anger and disgust ratings were higher than any other rating, suggesting that these expressions were perceived as somewhat mixed.

For happy and sad expressions, ambience only had an effect on the target emotions which were rated higher in a pleasant environment. No effect emerged for disgust expressions, whereas anger expressions were rated as more surprised when encountered in a pleasant ambience. For the full analyses see supplementary materials.

### Perceived closeness

Main effects of Expression, F(1, 1587) = 1257.45, *p* < .001, eta_p_^2^ = 0.442, and Ambience, F(1, 1578) = 7.97, *p* = .008, eta_p_^2^ = 0.005, emerged. The latter was qualified by the interaction, F(1, 1584) = 9.77, *p* = .002, eta_p_^2^ = 0.006. Overall, participants reported feeling closer to happy (M_pleasant_ = 37.80, SD = 22.39, CI_95_[35.64,39.96]; M_unpleasant_ = 34.11, SD = 20.48, CI_95_[32.12,36.10]) than to distressed avatars (M_pleasant_ = 11.30, SD = 13.20, CI_95_[10.02,12.58]; M_unpleasant_ = 10.67, SD = 12.94, CI_95_[9.42,11.92]). As expected, positive ambience increased closeness to happy avatars significantly, but the mean difference for distressed avatars was not significant. For the full analysis see supplementary materials.

## Discussion

The present study aimed to investigate how ambience influences empathy as assessed through emotional mimicry, emotion recognition and perceived interpersonal closeness. The findings support our core assumption that ambience has an impact on emotional mimicry, a physiological indicator of affiliation^16,17^ and affective empathy^20^.

Specifically, the pattern of facial responses is indicative of two influences. First, the ambience in which the expressions were shown induced a corresponding mood. In the negative ambience condition, this resulted in a corresponding negative expression which persisted during the time when participants saw the avatar with a neutral face. This already present expression then conflicted with the onset of happiness mimicry. As a result participants did not show happiness during the onset phase (as they did in the positive ambience condition) and the expression at apex was significantly less intense. Conversely, distress mimicry was strengthened by the persistent negative affect. As a result, both positive and negative ambience had a dampening effect on mimicry of the respective incongruent expression. The latter finding is consistent with the congruency effect generally observed for emotion communication. The affect induced by the positive ambience was less persistent yet seemed to slow down mimicry of distress faces.

The finding that mimicry was clearly evident in both ambience conditions is interesting in that it contrasts with research on congruent and incongruent social contexts. There, expressions that do not match the social context – that are socially deviant^44^– are often not mimicked at all^8^. By contrast, ambience, which is not clearly related to the emotion elicitor, had a more subtle dampening effect on mimicry responses. In fact, it seems that bottom-up affect induction effects and top-down congruency effects acted simultaneously. Thus, the findings for perceived closeness, which was only increased for happy expressions in the pleasant condition, were also in line with the congruency hypothesis.

By contrast, the pattern of results for emotion recognition was more in line with the empathy resources hypothesis. Although ambience had only a limited effect on emotion perception, the differences that did emerge suggest that a positive ambience increases emotion recognition for both happy and sad expressions.

One reason for this difference in findings might be that mimicry relates to affective empathy. By contrast, emotion ratings were obtained by ratings which require cognitive empathy^19^. Thus, these might have in fact profited from the broaden-and-build effect of a positive ambience, albeit only to a small degree.

In sum, the present research provides strong evidence that the ambience in which we encounter others affects the ensuing emotion communication. Notably, avatars encountered in a positive ambience were mimicked more when they showed a happy expression and those encountered in a negative ambience were mimicked more when showing a negative expression. Interestingly, in recent years, evidence has accumulated that whereas mimicry of affiliative expressions such as happiness is always associated with positive consequences, mimicry of antagonistic expressions such as anger or disgust is not and may even worsen interaction quality^45^. As such, this finding suggests that when we encounter someone in a negative context such as the construction site area of the park, an existing antagonism may actually be enhanced. By contrast, a pleasant environment fosters the positive effects of a smile. Effects on emotion recognition were more subtle, suggesting, if anything, that cognitively more demanding judgments may be, very slightly, enhanced by a positive ambience.

### Limitations

To the best of our knowledge, this is the first study that combined high-resolution VR technology and facial EMG to study how environmental ambience shapes affective and cognitive empathy. However, the study has also certain limitations. First, to be able to create a fully immersive virtual reality environment we had to use avatars. These were of very high quality but still may not have fully captured the emotional nuances of human faces. That said, previous research has shown that people mimic avatars in line with reactions to humans^46^. Further, our VR scenario overall lacked the complexities of real-world interactions, such as spontaneous conversation and non-verbal cues beyond facial expressions (e.g., voice). Thus, the situation remained artificially constrained, limiting generalizability to real contexts. At the same time, the immersive VR setup offered greater ecological validity than conventional screen-based laboratory paradigms, as participants encountered embodied avatars within a surrounding environment rather than viewing isolated facial stimuli detached from a situational context.

Finally, even though integrated dry-sensor EMG allows facial muscle activity to be recorded during immersive VR exposure where conventional electrodes may interfere with headset fit, this setup differs in sensor placement constraints, contact stability and potential movement artifacts. Yet, we monitored signal quality continuously and found that EMG results at the level of theoretically predicted muscle patterns can be interpreted in line with data collected using standard lab-based EMG systems.

## Conclusions

Taken together, the present study could show that emotional mimicry is informed by ambience information in VR environments. This is relevant because mimicry has an important influence on the quality of interactions^45^. Further, the findings point toward a potential novel route for emotion communication research, whereby the ambience or “vibe” of a situation can be studied in immersive scenarios.

The present research aligns with an increased empirical focus on “embedded cognition” that has attempted to address the lack of research that has considered the inherently situated nature of being. This work can be considered part of the multiple E approaches to cognition, which includes 4E cognition (embodied, embedded, enactive, and extended^47^) by providing new avenues to study the impact of embeddedness. Finally, from a simple methodological perspective, embedded approaches have the advantage of increased ecological validity.

To conclude, the present research showed that an automatic, albeit goal dependent, index of affective empathy – emotional mimicry – is moderated by the ambience of the setting in which an expression is encountered. This novel and unique evidence contributes to a deeper understanding of how emotional processes are embedded in the physical and social worlds that provide affective ambiences for social interactions. While maybe not surprising, this research shows why planning a first date at a construction site is probably not the best idea.

## Methods

### Participants

We recruited a total of 78 German-speaking individuals. Based on a power simulation (see below) we aimed for 70 participants but oversampled to compensate for possible data loss. Mimicry data from three participants had to be excluded due to technical problems resulting in a final sample of *N* = 75 (51 women, 21 men, and 3 diverse gender identities) with a mean age of 28 years (SD = 7). All participants had normal or corrected-to-normal vision (i.e., contact lenses or glasses that fit under the headset).

### Design, sample size justification, and power analysis

The experiment followed a 2 ambience (pleasant vs. unpleasant) x 2 expressions (happy vs. distress) design. The selected minimal sample size of 70 participants was determined based on economic considerations and supported by a *power simulation* conducted in R (simr^48^) for a 2 * 2 design assuming effect sizes based on similar studies conducted in the laboratory using video material. A sensitivity analysis based on the current sample size confirmed adequate power to detect an effect of Cohen’s f = 0.025 (see supplementary materials).

### Materials

#### Virtual reality environments

Using Unreal Engine 5.0 by Epic Games, we created two virtual environments (see https://osf.io/f6vht for a rendered video of the surroundings) situated in a city park: a tranquil and picturesque area with greenery and bird song (pleasant ambience) and a bustling construction site with construction noise (e.g., vehicles, drills, moving machines) within another location of the park (unpleasant ambience). The environments were pretested with an independent sample (*N* = 100, 48 women, 49 men, 3 diverse). An additional manipulation check for the places was conducted at the end of the study (see results section and supplementary materials). The pleasant ambience aimed to foster positive affect and tranquility. This place featured sakuras (cherry trees), fountains, monuments, artworks, a vibrant café, street musicians (playing Schubert’s Ständchen in C major), the latter rated as pleasant with a mean of 83.18, *SD* = 15.72; on a pleasantness scale from 1 to 101), a public piano (with someone playing Schumann’s Träumerei in F major; faces of musicians not visible, located in the distance), rated as pleasant with a mean of 82.82, *SD* = 16.54, and a vintage carousel (own music production), rated as pleasant with a mean of 67.34, *SD* = 21.41.

By contrast, the unpleasant ambience was intended to induce stress. This place featured rubble, portable toilets, construction vehicles, an ambulance helicopter flying over the scene, the latter rated as unpleasant with a mean of 25.93, *SD* = 16.46, two ambulance cars arriving with siren on, rated as unpleasant with a mean of 17.10, *SD* = 14.81, and a descending jumbo jet approaching a nearby airport, rated as unpleasant with a mean of 23.93, *SD* = 17.60. Environmental sound assets were licensed from Envato Elements and merged to two separate soundscapes in Ableton Live 11, the music was bought from Qobuz or produced with Ableton Live 11 (the sound assets can be found on OSF).

### Avatars and emotion expressions

Using MetaHuman Creator by Epic Games, we created diverse, realistic avatars (male and female with varying hair colors and skin tones) with nuanced emotion expressions (see Fig. 1), based on the templates provided by Unreal animations, which are guided by but not fully correspond to the terminology of the Facial Action Coding System^49^. In the present study, we use the term “avatar” to refer to computer-generated, human-like digital representations presented as stimuli in the VR environment; this usage does not imply real-time control by another human participant, nor autonomous agency of the characters. We introduced the avatars to participants as digital representations of humans (for full details of the verbal instructions given to participants, see supplementary materials in OSF). Following the experiment, participants were asked to rate the human-likeness of the avatars on a scale from 1 - not at all human-like to 101 - very human-like. Overall, avatars were perceived as moderately humanlike (*M* = 61.73, *SD* = 22.67). We also asked about the realism of the avatars in the debriefing interview, which returned similar results with a mean realism rating of *M* = 69.28 (*SD* = 17.91).

### Perception of the VR setting

Forty-nine participants (i.e. 65.3%) reported that they had used a virtual reality headset before, the most frequently mentioned domains were experiments^31^, games^17^, and museums^4^. Mean pupil distance was 5.98 cm (*SD* = 0.4). The eye adjustment score in the headset was 64.98 mm (*SD* = 1.9).

VR approval ratings on 0 to 100 scales were 71.6 (*SD* = 22.6) for headset comfort, 67.0 (*SD* = 21.95) for headset sight, 94.3 (*SD* = 15.3) for ease in controller use, and 61.19 (*SD* = 25.0) for similarity of the VR environment to the real world. Only 14 participants reported slight delays in controller actions and the expected outcomes of these actions. Even fewer participants reported (mild) motion sickness in the domains of sense of balance^3^ and nausea^5^, both of which, however, did not cause severe problems as indicated by debriefings and non-use of the experiment interruption button.

### Dependent variables

*Emotional mimicry* was assessed by measuring the activity of the Orbicularis Oculi lateralis (the muscle that creates the wrinkles around the eye in smiling), Zygomaticus Major (the muscle that pulls the corner of the lips up), and Corrugator Supercilii (the muscle that pulls the eyebrows down in a frown) using the integrated EMG in emteqPRO^39^. This recording system has been successfully validated in previous studies that investigated facial responses to affective stimuli such as voluntary mimicry responses to emotion expressions^50^. Moreover, the system has been found to be capable of measuring both posed, voluntary facial movements and spontaneous muscle activations while users view pre-validated affective video clips^39,51^. Further, recordings from the system have been utilized to train machine learning models capable of predicting classes of activity such as valence or discrete facial expressions^52–54^.

Participants were instructed to not apply makeup and if needed to shave the area of the Zygomaticus M. electrode site. Skin was prepared using alcohol swabs to remove skin oils. Impedances were below 25k Ω. The signal was recorded with 1000 Hz. The raw EMG signal was notch-filtered with a 50 Hz and bandpass-filtered with the predefined filtering algorithm provided by emteq with a range of 100 to 450 Hz, rectified, and smoothed. Data were baseline-corrected (pre-stimulus baseline) and within-subjects z-standardized^55^ in R using base R^43^ and the *tidyverse*^56^.

*Emotion perception* was measured using an emotion profile that assessed six emotions: happiness, sadness, fear, anger, disgust, and surprise. Each emotion was rated on a scale ranging from 1-not at all to 7-very much.

*Perceived interpersonal closeness*, or self-other overlap, was measured using an adapted version of the Inclusion of Other in the Self (IOS) scale. Participants adjusted the relative position of a circle representing themselves to a circle representing the avatar using a slider on a scale from 1 to 101 to represent the degree of self-other overlap, which is a way to operationalize constructs such as psychological closeness, social connection, or, put more generally, affiliation.

### Procedure

Participants were welcomed to the virtual reality laboratory and asked to sit in a comfortable armchair. Participants provided informed consent (including an explanation about the use of sensor technology) and were briefed about the participant rights provided by the General Data Protection Regulation (GDPR) of the European Union (EU). The study was conducted in accordance with the Declaration of Helsinki and the Guidelines for Safeguarding Good Research Practice of the German Research Foundation. The study was approved by the Institutional Review Board of the Department of Psychology (#2018-20).

The experimenter fitted participants with HTC Vive Pro Eye virtual reality head-mounted displays (HMD). The HMD employed SteamVR 2.7.4 running on a PCGH Ultimate-PC 2080Ti-Edition (Windows 10 Pro) with an Intel^®^ Core™ i9-9900 K processor and an NVIDIA GeForce RTX 2080 Ti graphics processing unit. Local data storage consisted of two drives with capacities of approximately 1 TB and 4 TB, respectively. The HMD was equipped with an emteqPRO inlay with integrated emteq electromyography (EMG) dry sensors and photoplethysmography (PPG). Depending on the face shape of participants, the wide or narrow mask system was used.

After fitting the headset, the experimenter checked sensor contact and signal quality in real time using the emteq SuperVision application. The headset position and strap tightness were adjusted until stable signals were visible for the relevant channels. The data was monitored in real-time using the emteq OpenFaceSystem 1.5.4.6 (a Unity-based SDK) and the emteq SuperVision recording and monitoring 1.4.0 application. After each data collection, the emg data were processed via the Data Insights tool in the SuperVision software before being used for external processing via PhysioData Toolbox^57^ based on MATLAB. The data stream from these systems was merged with the markers from the Unreal procedure via a pseudonym participant code as well as time stamps based on the UNIX-2000 time using the Unreal plugins easyCSV and Runtime DataTable.

Participants completed a 2-minute baseline and a subsequent calibration of the headset. During the 2-minute baseline, participants were asked to rest with no further instructions given (during that time they saw the night sky with stars of the SteamVR home environment). This baseline is part of the standard setup of the headset. The collected baseline values were not used in our analyses. The baseline values of the EMG baseline measures were taken from a trial-based, pre-stimulus baseline.

The experimenter read the instructions which included information about the walk-in-the-park scenario and the concept of avatars. Specifically, we briefed participants with a verbal instruction that could be translated as follows: “We are particularly interested in the very first moment of encountering a previously unknown person. We therefore ask you to imagine as vividly as possible that you are moving through this virtual environment and meeting people there who approach you and might speak to you or ask you something in the next moment. It’s important to note, though, that this act of being approached, and the possibility of a conversation, is not the goal of the experiment – the focus is explicitly on the moment just before that. We find this a fascinating moment: the moment when you meet someone new for the first time in your life, when at first there is only nonverbal interaction. We ask you to focus on this moment. At the same time, please remain aware of where you are. We ask you to immerse yourself as fully as possible in the location and the people there.” For full details on the verbal instructions, see supplementary material in OSF. Participants familiarized themselves with the VR equipment before being randomly assigned to either a pleasant-unpleasant or unpleasant-pleasant order.

The ambience conditions were blocked with 12 trials per block (six happy expressions, and two each anger, sadness and disgust expressions). Each trial started with a pre-stimulus fixation cross shown for 1.5 s (sec) on a blackboard that appeared in the park which served as pre-stimulus data baseline for the EMG data. The stimulus consisted of 7.5 s of the park scene, before another fixation cross (1.5 s) was shown. Then the avatar was presented at 200 cm distance away from participants (Unreal allows a true-to-scale distance in VR) showing first a neutral expression (1.5 s), followed by a 2 s onset phase, 1.5 s at apex and concluding with a post stimulus blackboard embedded into the space (1.5 s). Participants were asked to watch the stimuli and provided ratings after each encounter. For this, following each encounter, participants saw blackboards embedded into the scene that included the emotion profile for the perceived emotion ratings and the interpersonal closeness scale. They provided ratings by using the laser pointer of the VR controller before moving on to the next trial. Participants were not allowed to speak during the experiment but could interrupt the experiment during each rating phase via a call button in case of questions or problems; none of the participants used this button.

Following the experiment, participants provided demographic data and completed a questionnaire with ratings of the VR environment. Participants were then thoroughly debriefed. The debriefing interview was transcribed and analyzed using the *sentimentr* package^58^. The sentiment score was positive (0.36). Participants used 84% positive terms with ‘cool’ and ‘exciting’ being the most frequently mentioned terms vs. 16% negative terms with ‘exhausting’ and ‘difficult’ being mentioned most frequently. These qualitative insights are particularly interesting given that the experiment also had a clearly unpleasant condition. At the conclusion of the study, participants were compensated, either with 10 € or course credit, thanked for their participation and dismissed. The session time (i.e., briefing, experiment, debriefing) average was 55 min and the mean experiment time 28 min.

## Supplementary Information

Below is the link to the electronic supplementary material.

Supplementary Material 1

## Data Availability

The data for this study and a complete markdown are available at OSF (https://osf.io/vcnp4/files/osfstorage).
