# Supplementary material for: Environmental context effects on affective and cognitive empathy in virtual reality
Source: Sci Rep. 2026 Aug 14;16:25476. doi: 10.1038/s41598-026-66551-9 (PMC13476244; doi:10.1038/s41598-026-66551-9)
Supplement: Supplementary file 1 [file 41598_2026_66551_MOESM1_ESM.pdf]

Results for analyses reported in the text

2026-08-03

Contents

1 Basic EMG Analyses **2**
- 1.1 Plot full model . . . 2
- 1.2 Analyses . . . 3
- 1.3 Happy Expression . . . 3
- 1.4 Distress expression . . . 3

2 Analyses apex-neutral **4**

3 Rating Data **4**
- 3.1 Emotion recognition: Graph emotion profile . . . 4
- 3.2 Emotion recognition: Full model . . . 4
- 3.3 Emotion Recognition: Post-hoc tests . . . 5
  - 3.3.1 Happy Expressions . . . 5
  - 3.3.2 Sad expressions . . . 5
  - 3.3.3 Anger expressions . . . 6
  - 3.3.4 Anger expressions . . . 7
- 3.4 Contrasts for ambience effects across scales . . . 7
- 3.5 IOS . . . 8

4 Manipulation check **9**

5 Sensitivity Analysis **10**


```
## Finding R package dependencies ... Done!
```

Table 1: Packages used in this analysis

	Package	Version
rmarkdown	rmarkdown	2.29
car	car	3.1.3
dplyr	dplyr	1.1.4
DT	DT	0.33
effectsize	effectsize	1.0.1
emmeans	emmeans	1.11.2
ggeffects	ggeffects	2.3.0
ggplot2	ggplot2	4.0.3
ggpubr	ggpubr	0.6.1
ggsci	ggsci	3.2.0
haven	haven	2.5.5
kableExtra	kableExtra	1.4.0
knitr	knitr	1.50
lavaan	lavaan	0.6.21
lavaanPlot	lavaanPlot	0.8.1
lme4	lme4	1.1.37
lmerTest	lmerTest	3.1.3
misty	misty	0.7.3
paletteer	paletteer	1.6.0
psych	psych	2.5.6
rstatix	rstatix	0.7.2
sjPlot	sjPlot	2.9.0
sjstats	sjstats	0.19.1
stringr	stringr	1.5.1
tidyr	tidyr	1.3.1
tidyverse	tidyverse	2.0.0
utils	utils	4.5.2
renv	renv	1.2.0
rstudioapi	rstudioapi	0.17.1
ggthemes	ggthemes	5.2.0

1 Basic EMG Analyses

1.1 Plot full model

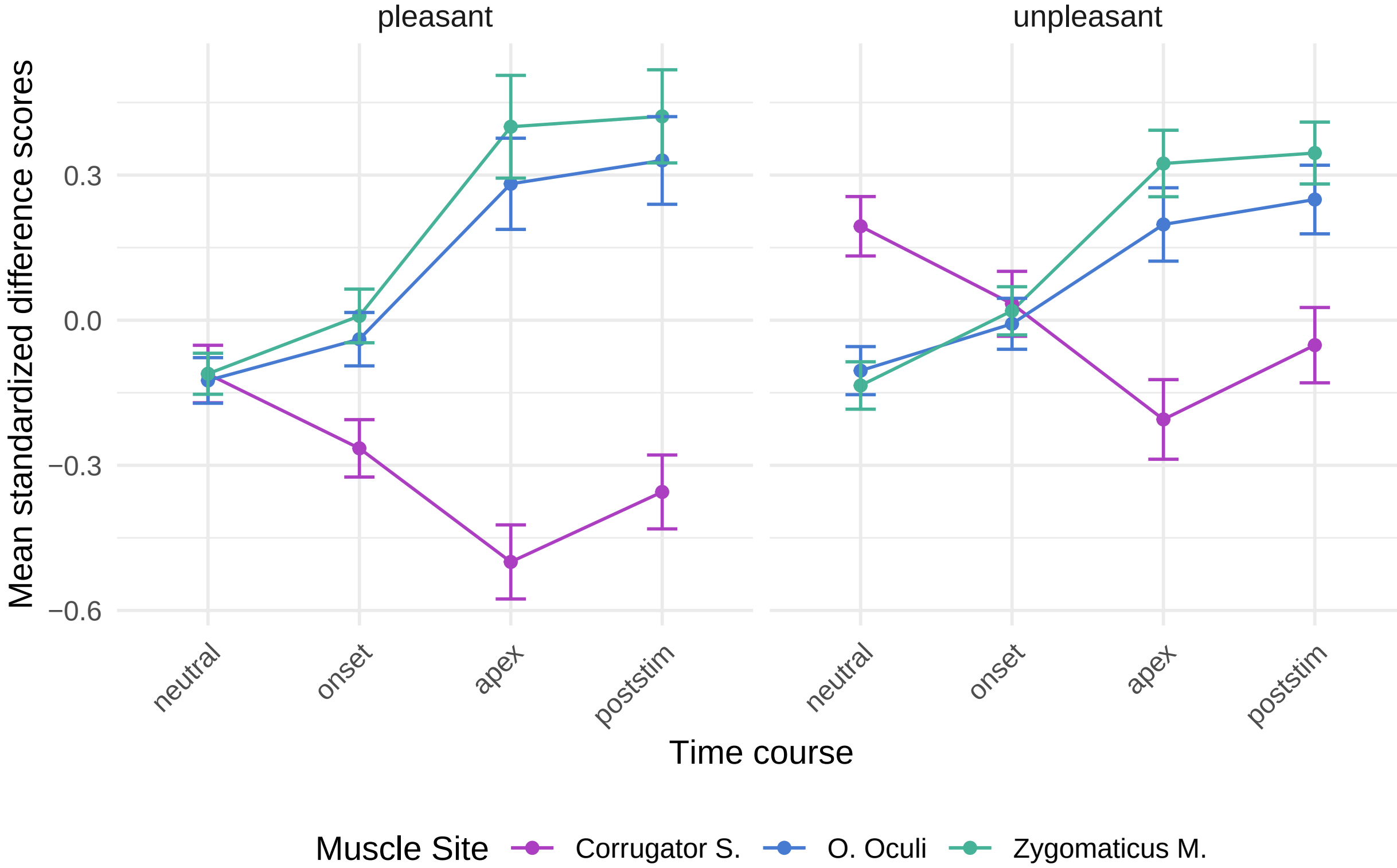

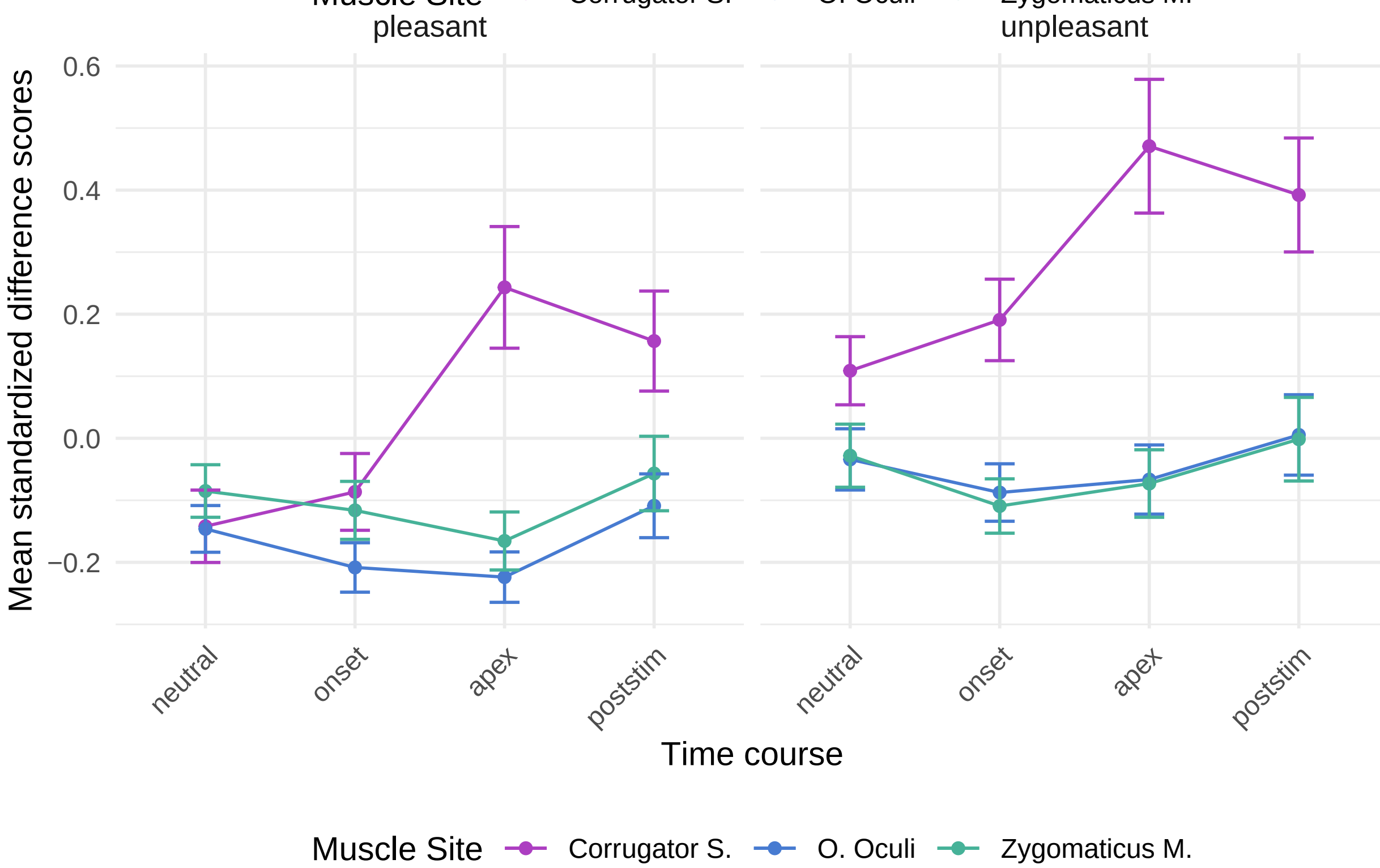

1.2 Analyses

Helmert contrast between Corrugator S. and mean of O. Oculi and Zygomaticus M. for each segment and ambience. Mimicry is present when the contrast is significant and negative.

A-priori contrasts for ambience: significant contrast indicates that mimicry intensity differs between ambiences

1.3 Happy Expression

Table 2: ANOVA Results with Partial Eta Squared

	Sum Sq	Mean Sq	NumDF	DenDF	F value	Pr(>F)	Partial ^{2}
segment	72.440	24.147	3	9845.998	58.251	0.000	0.017
muscle	192.335	96.168	2	9870.793	231.996	0.000	0.045
Ambience	9.230	9.230	1	9916.349	22.268	0.000	0.002
segment:muscle	198.274	33.046	6	9845.513	79.720	0.000	0.046
segment:Ambience	0.207	0.069	3	9845.889	0.166	0.919	0.000
muscle:Ambience	49.252	24.626	2	9849.663	59.408	0.000	0.012
segment:muscle:Ambience	1.170	0.195	6	9845.138	0.470	0.831	0.000

Table 3: Helmert Contrast by Segment and Ambience

**contrast**	**segment**	**Ambience**	**estimate**	**SE**	**df**	**t.ratio**	**p.value**
muscle	neutral	pleasant	0.067	0.077	9852.469	0.864	0.388
muscle	onset	pleasant	0.559	0.077	9852.112	7.237	0.000
muscle	apex	pleasant	1.571	0.078	9854.281	20.198	0.000
muscle	poststim	pleasant	1.367	0.078	9854.758	17.503	0.000
muscle	neutral	unpleasant	-0.523	0.078	9856.843	-6.687	0.000
muscle	onset	unpleasant	0.021	0.078	9855.376	0.270	0.787
muscle	apex	unpleasant	0.967	0.078	9856.066	12.373	0.000
muscle	poststim	unpleasant	0.700	0.078	9857.038	8.952	0.000

Table 4: A priori contrasts for Ambience

**contrast**	**segment**	**estimate**	**SE**	**df**	**t.ratio**	**p.value**
muscle unpleasant - muscle pleasant	neutral	-0.590	0.110	9848.725	-5.368	0
muscle unpleasant - muscle pleasant	onset	-0.538	0.109	9848.232	-4.914	0
muscle unpleasant - muscle pleasant	apex	-0.604	0.110	9849.170	-5.489	0
muscle unpleasant - muscle pleasant	poststim	-0.667	0.110	9848.938	-6.039	0

1.4 Distress expression

A-priori contrasts for ambience: significant contrast indicates that mimicry intensity differs between ambiences

Table 5: ANOVA Results with Partial Eta Squared

	Sum Sq	Mean Sq	NumDF	DenDF	F value	Pr(>F)	Partial ^{2}

segment	21.473	7.158	3	10362.38	18.769	0.000	0.005
muscle	163.946	81.973	2	10383.39	214.949	0.000	0.040
Ambience	53.099	53.099	1	10433.93	139.236	0.000	0.013
segment:muscle	50.231	8.372	6	10362.22	21.953	0.000	0.013
segment:Ambience	0.907	0.302	3	10362.00	0.793	0.498	0.000
muscle:Ambience	20.951	10.475	2	10366.81	27.469	0.000	0.005
segment:muscle:Ambience	0.595	0.099	6	10361.96	0.260	0.955	0.000

Table 6: Helmert contrast as a function of Segment and Ambience

**contrast**	**segment**	**Ambience**	**estimate**	**SE**	**df**	**t.ratio**	**p.value**
muscle	neutral	pleasant	-0.011	0.072	10370.19	-0.148	0.882
muscle	onset	pleasant	-0.142	0.072	10370.46	-1.955	0.051
muscle	apex	pleasant	-0.796	0.074	10370.53	-10.804	0.000
muscle	poststim	pleasant	-0.484	0.074	10370.48	-6.564	0.000
muscle	neutral	unpleasant	-0.330	0.073	10371.66	-4.515	0.000
muscle	onset	unpleasant	-0.609	0.073	10371.59	-8.301	0.000
muscle	apex	unpleasant	-1.120	0.074	10371.81	-15.190	0.000
muscle	poststim	unpleasant	-0.831	0.074	10370.57	-11.233	0.000

Table 7: A priori contrasts for Ambience

**contrast**	**segment**	**estimate**	**SE**	**df**	**t.ratio**	**p.value**
muscle unpleasant - muscle pleasant	neutral	-0.320	0.103	10366.80	-3.109	0.002
muscle unpleasant - muscle pleasant	onset	-0.468	0.103	10367.16	-4.535	0.000
muscle unpleasant - muscle pleasant	apex	-0.325	0.104	10366.95	-3.119	0.002
muscle unpleasant - muscle pleasant	poststim	-0.348	0.104	10366.46	-3.333	0.001

2 Analyses apex-neutral

Table 8: Descriptive statistics for difference scores.

Expression	Ambience	neutral	onset	apex	poststim	Difference
distress	pleasant	0.03 (0.53)	-0.07 (0.59)	-0.44 (0.88)	-0.25 (0.87)	-0.502 (0.93)
distress	unpleasant	-0.15 (0.66)	-0.31 (0.72)	-0.54 (1.08)	-0.43 (1.03)	-0.404 (0.93)
joy	pleasant	-0.03 (0.60)	0.24 (0.75)	0.83 (1.32)	0.77 (1.15)	0.904 (1.19)
joy	unpleasant	-0.33 (0.64)	-0.04 (0.74)	0.46 (1.01)	0.33 (0.90)	0.817 (1.10)

3 Rating Data

3.1 Emotion recognition: Graph emotion profile

3.2 Emotion recognition: Full model

Table 9: ANOVA Results with Partial Eta Squared

	Sum Sq	Mean Sq	NumDF	DenDF	F value	Pr(>F)	Partial ^{2}
EmoScale	7073390.043	1414678.009	5	122392.0	5434.784	0.000	0.182
Expression	409942.518	409942.518	1	122429.5	1574.881	0.000	0.013
Ambience	5498.346	5498.346	1	122394.0	21.123	0.000	0.000
EmoScale:Expression	25676908.376	5135381.675	5	122392.0	19728.650	0.000	0.446
EmoScale:Ambience	45247.825	9049.565	5	122392.0	34.766	0.000	0.001
Expression:Ambience	2.917	2.917	1	122425.3	0.011	0.916	0.000
EmoScale:Expression:Ambience	50220.045	10044.009	5	122392.0	38.586	0.000	0.002

3.3 Emotion Recognition: Post-hoc tests

To follow up on the significant effects, contrast were calculated comparing the rating on the tagert scale with the ratings on all other scales.

3.3.1 Happy Expressions

Table 10: ANOVA Results with Partial Eta Squared

	Sum Sq	Mean Sq	NumDF	DenDF	F value	Pr(>F)	Partial ^{2}
EmoScale	25446894	5089379	5	59596.06	40151.98	0	0.771

Table 11: Happy Expressions: Rated Happiness vs. other

**contrast**	**estimate**	**SE**	**df**	**z.ratio**	**p.value**
sad - happy	-55.760	0.16	Inf	-349.266	0
anger - happy	-57.063	0.16	Inf	-357.426	0
fear - happy	-55.658	0.16	Inf	-348.625	0
disgust - happy	-57.568	0.16	Inf	-360.586	0
surprise - happy	-46.237	0.16	Inf	-289.615	0

3.3.2 Sad expressions

Table 12: ANOVA Results with Partial Eta Squared

	Sum Sq	Mean Sq	NumDF	DenDF	F value	Pr(>F)	Partial ^{2}
EmoScale	5001095	1000219	5	20067.99	3895.322	0	0.493

Table 13: Sad Expressions: Rated Sadness vs. other

**contrast**	**estimate**	**SE**	**df**	**z.ratio**	**p.value**
happy - sad	-48.228	0.391	Inf	-123.323	0
anger - sad	-33.307	0.391	Inf	-85.171	0
fear - sad	-38.819	0.391	Inf	-99.264	0
disgust - sad	-39.859	0.391	Inf	-101.925	0
surprise - sad	-42.550	0.391	Inf	-108.806	0

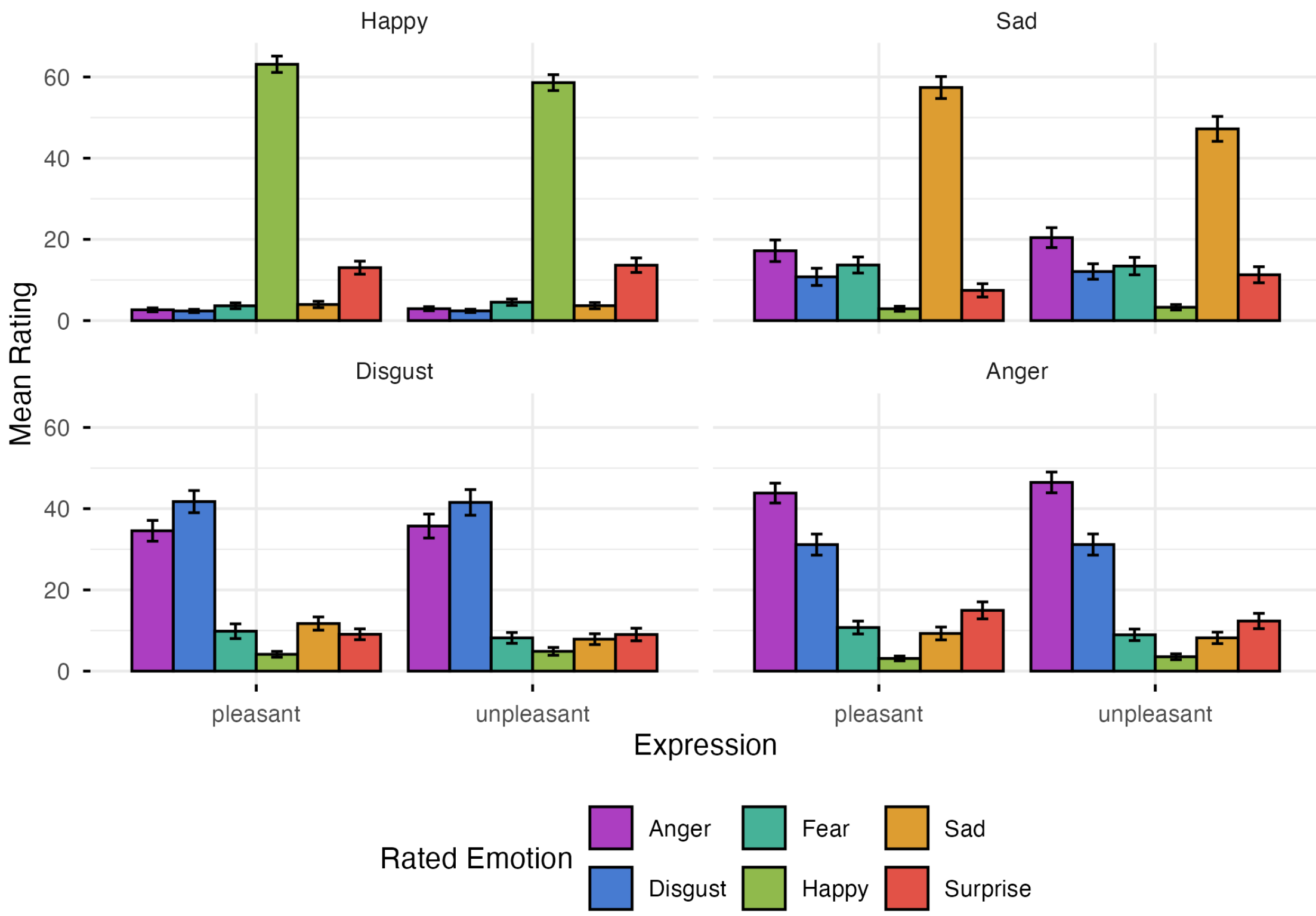

Figure 1: Mean emotion ratings by expression and condition.

3.3.3 Anger expressions

Table 14: ANOVA Results with Partial Eta Squared

	Sum Sq	Mean Sq	NumDF	DenDF	F value	Pr(>F)	Partial ^{2}
EmoScale	4603689	920737.9	5	21850.07	3509.165	0	0.445

Table 15: Anger Expressions: Rated Anger vs. other

**contrast**	**estimate**	**SE**	**df**	**z.ratio**	**p.value**
happy - anger	-41.071	0.379	Inf	-108.393	0
sad - anger	-35.904	0.379	Inf	-94.756	0
fear - anger	-33.990	0.379	Inf	-89.706	0
disgust - anger	-12.816	0.379	Inf	-33.824	0
surprise - anger	-30.594	0.379	Inf	-80.741	0

Table 16: Anger Expressions: Rated Disgust vs. other

**contrast**	**estimate**	**SE**	**df**	**z.ratio**	**p.value**
happy - disgust	-28.255	0.379	Inf	-74.570	0
sad - disgust	-23.088	0.379	Inf	-60.932	0
anger - disgust	12.816	0.379	Inf	33.824	0
fear - disgust	-21.174	0.379	Inf	-55.882	0
surprise - disgust	-17.778	0.379	Inf	-46.917	0

3.3.4 Anger expressions

Table 17: ANOVA Results with Partial Eta Squared

	Sum Sq	Mean Sq	NumDF	DenDF	F value	Pr(>F)	Partial ^{2}
EmoScale	4112365	822472.9	5	20656.8	2936.335	0	0.415

Table 18: Disgust Expressions: Rated Disgust vs. other

**contrast**	**estimate**	**SE**	**df**	**z.ratio**	**p.value**
happy - disgust	-36.095	0.403	Inf	-89.653	0
sad - disgust	-30.794	0.403	Inf	-76.486	0
anger - disgust	-6.131	0.403	Inf	-15.229	0
fear - disgust	-31.604	0.403	Inf	-78.498	0
surprise - disgust	-31.411	0.403	Inf	-78.018	0

Table 19: Disgust Expressions: Rated Anger vs. other

**contrast**	**estimate**	**SE**	**df**	**z.ratio**	**p.value**
happy - anger	-29.964	0.403	Inf	-74.424	0
sad - anger	-24.663	0.403	Inf	-61.257	0
fear - anger	-25.473	0.403	Inf	-63.269	0
disgust - anger	6.131	0.403	Inf	15.229	0
surprise - anger	-25.280	0.403	Inf	-62.789	0

3.4 Contrasts for ambience effects across scales

Table 20: ANOVA Results with Partial Eta Squared

	Sum Sq	Mean Sq	NumDF	DenDF	F value	Pr(>F)	Partial ^{2}
EmoScale	3006899.563	601379.913	5	122368.0	2959.340	0	0.108
Exp_cat	424215.755	141405.252	3	122393.9	695.843	0	0.017
Ambience	4133.462	4133.462	1	122375.1	20.340	0	0.000
EmoScale:Exp_cat	32586510.129	2172434.009	15	122368.0	10690.365	0	0.567
EmoScale:Ambience	74494.890	14898.978	5	122368.0	73.317	0	0.003
Exp_cat:Ambience	5361.878	1787.293	3	122393.5	8.795	0	0.000
EmoScale:Exp_cat:Ambience	113519.498	7567.967	15	122368.0	37.241	0	0.005

Table 21: Pairwise Contrasts (Pleasant vs. Unpleasant Perception) by Emotion Scale and Expression Category.

Emotion Scale	Expression Category	Estimate	SE	df	z ratio	p value
happy	hap	5.310	0.287	Inf	18.535	<.001
happy	sad	0.407	0.495	Inf	0.823	0.411
happy	dis	-0.526	0.488	Inf	-1.079	0.281
happy	ang	0.007	0.473	Inf	0.014	0.989
sad	hap	0.265	0.287	Inf	0.924	0.356
sad	sad	8.643	0.495	Inf	17.475	<.001
sad	dis	3.008	0.488	Inf	6.168	<.001
sad	ang	2.057	0.473	Inf	4.344	<.001
anger	hap	-0.682	0.287	Inf	-2.382	0.017
anger	sad	-3.040	0.495	Inf	-6.146	<.001
anger	dis	-2.095	0.488	Inf	-4.297	<.001
anger	ang	-1.860	0.473	Inf	-3.928	<.001
fear	hap	-0.999	0.287	Inf	-3.487	<.001
fear	sad	0.744	0.495	Inf	1.505	0.132
fear	dis	0.046	0.488	Inf	0.094	0.925
fear	ang	2.838	0.473	Inf	5.994	<.001
disgust	hap	-0.535	0.287	Inf	-1.866	0.062
disgust	sad	-1.571	0.495	Inf	-3.176	0.001
disgust	dis	-1.486	0.488	Inf	-3.047	0.002
disgust	ang	-0.311	0.473	Inf	-0.657	0.511
surprise	hap	-0.887	0.287	Inf	-3.095	0.002
surprise	sad	-2.574	0.495	Inf	-5.205	<.001
surprise	dis	-0.927	0.488	Inf	-1.901	0.057
surprise	ang	4.003	0.473	Inf	8.457	<.001

Note: Contrast estimates represent 'pleasant - unpleasant'. Degrees-of-freedom method: kenward-roger.

3.5 IOS

Table 22: ANOVA Results with Partial Eta Squared

	Sum Sq	Mean Sq	NumDF	DenDF	F value	Pr(>F)	Partial ^{2}
Ambience	20528.65	20528.65	1	20337.53	114.046	0	0.006
Expression	2900908.53	2900908.53	1	20348.31	16115.933	0	0.442
Ambience:Expression	18669.35	18669.35	1	20346.85	103.717	0	0.005

Table 23: Difference between ambiences as a function of expression for IOS

**contrast**	**Expression**	**estimate**	**SE**	**df**	**z.ratio**	**p.value**
pleasant - unpleasant	distress	0.049	0.266	Inf	0.185	0.854
pleasant - unpleasant	joy	3.968	0.273	Inf	14.560	0.000

Table 24: **Means and standard deviations for IOS**

Expression	Ambience	mean	sd	ci_lower	ci_upper

distress	pleasant	11.09	12.93	10.74	11.44
distress	unpleasant	10.44	12.84	10.09	10.79
joy	pleasant	37.18	22.23	36.56	37.80
joy	unpleasant	33.74	20.42	33.17	34.31

4 Manipulation check

Table 25: Emotion Ratings Summary

Mode	Mood	Emotion Type	N	Mean	SD	95% CI
Visual	Pleasant	Negative Emotions	78	2.96	4.76	[1.88, 4.03]
Visual	Pleasant	Positive Emotions	78	45.20	19.47	[40.81, 49.59]
Visual	Unpleasant	Negative Emotions	78	19.45	14.41	[16.2, 22.7]
Visual	Unpleasant	Positive Emotions	78	9.38	12.81	[6.49, 12.26]
Auditory	Pleasant	Negative Emotions	78	5.20	12.55	[2.37, 8.02]
Auditory	Pleasant	Positive Emotions	78	38.60	20.90	[33.89, 43.31]
Auditory	Unpleasant	Negative Emotions	78	21.70	15.40	[18.23, 25.18]
Auditory	Unpleasant	Positive Emotions	78	7.46	9.92	[5.22, 9.69]

Table 26: ANOVA Results with Partial Eta Squared

	Sum Sq	Mean Sq	NumDF	DenDF	F value	Pr(>F)	Partial ^{2}
Mode	450.594	450.594	1	1825.938	0.931	0.335	0.001
Mood	30423.571	30423.571	1	1827.438	62.846	0.000	0.033
EmoType	68412.054	68412.054	1	1825.938	141.318	0.000	0.072
Mode:Mood	574.173	574.173	1	1825.938	1.186	0.276	0.001
Mode:EmoType	4420.704	4420.704	1	1825.938	9.132	0.003	0.005
Mood:EmoType	264150.560	264150.560	1	1825.938	545.653	0.000	0.230
Mode:Mood:EmoType	575.404	575.404	1	1825.938	1.189	0.276	0.001

```
##
##
## |contrast               |EmoType |Mode     | estimate|    SE|       df| t.ratio| p.value| Cohen_s_d|
## |:----------------------|:-------|:--------|--------:|-----:|--------:|-------:|-------:|---------:|
## |Pleasant - Unpleasant  |negEmo  |Visual   |  -16.485| 1.745| 1823.622|  -9.445|       0|    -0.221|
## |Pleasant - Unpleasant  |posEmo  |Visual   |   35.766| 2.468| 1823.330|  14.492|       0|     0.339|
## |Pleasant - Unpleasant  |negEmo  |Auditory |  -16.482| 1.745| 1823.622|  -9.443|       0|    -0.221|
## |Pleasant - Unpleasant  |posEmo  |Auditory |   31.109| 2.468| 1823.330|  12.605|       0|     0.295|
```

5 Sensitivity Analysis

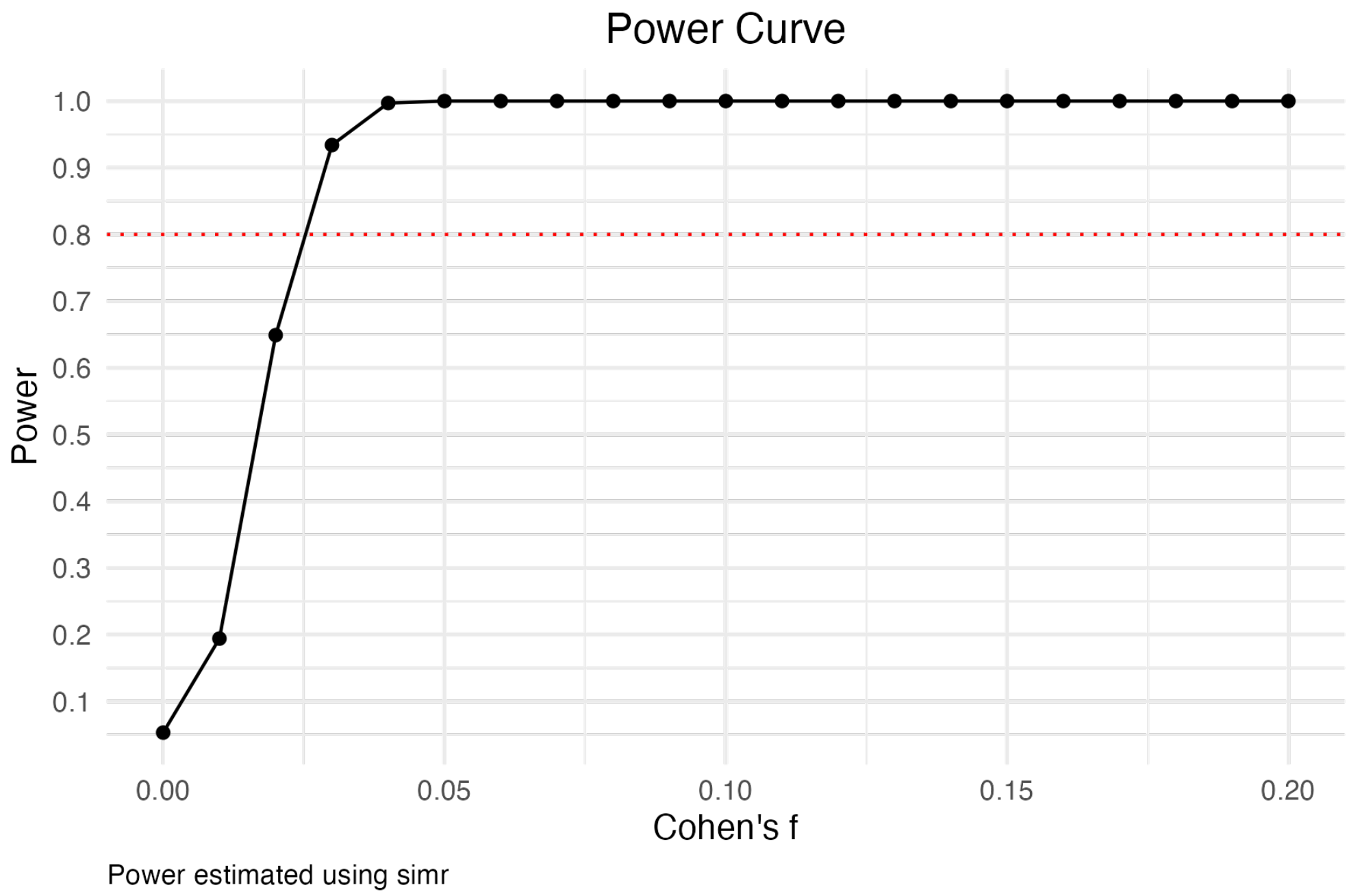

Table 27: Power Analysis Results

beta	power	lower	upper	successes	trials	warnings	errors	cohens_f
0.000	0.053	0.040	0.069	53	1000	0	0	0.00
0.398	0.194	0.170	0.220	194	1000	0	0	0.01
0.796	0.649	0.619	0.679	649	1000	0	0	0.02
1.195	0.934	0.917	0.949	934	1000	0	0	0.03
1.594	0.997	0.991	0.999	997	1000	0	0	0.04
1.993	1.000	0.996	1.000	1000	1000	0	0	0.05
2.393	1.000	0.996	1.000	1000	1000	0	0	0.06
2.793	1.000	0.996	1.000	1000	1000	0	0	0.07
3.195	1.000	0.996	1.000	1000	1000	0	0	0.08
3.597	1.000	0.996	1.000	1000	1000	0	0	0.09
4.001	1.000	0.996	1.000	1000	1000	0	0	0.10
4.406	1.000	0.996	1.000	1000	1000	0	0	0.11
4.812	1.000	0.996	1.000	1000	1000	0	0	0.12
5.219	1.000	0.996	1.000	1000	1000	0	0	0.13
5.629	1.000	0.996	1.000	1000	1000	0	0	0.14
6.040	1.000	0.996	1.000	1000	1000	0	0	0.15
6.452	1.000	0.996	1.000	1000	1000	0	0	0.16
6.867	1.000	0.996	1.000	1000	1000	0	0	0.17
7.284	1.000	0.996	1.000	1000	1000	0	0	0.18
7.704	1.000	0.996	1.000	1000	1000	0	0	0.19
8.126	1.000	0.996	1.000	1000	1000	0	0	0.20
